# DNA Methylation Profiles at Precancerous Stages Associated with Recurrence of Lung Adenocarcinoma

**DOI:** 10.1371/journal.pone.0059444

**Published:** 2013-03-27

**Authors:** Takashi Sato, Eri Arai, Takashi Kohno, Koji Tsuta, Shun-ichi Watanabe, Kenzo Soejima, Tomoko Betsuyaku, Yae Kanai

**Affiliations:** 1 Division of Molecular Pathology, National Cancer Center Research Institute, Tokyo, Japan; 2 Division of Pulmonary Medicine, Department of Medicine, Keio University School of Medicine, Tokyo, Japan; 3 Division of Genome Biology, National Cancer Center Research Institute, Tokyo, Japan; 4 Department of Pathology and Clinical Laboratories, Pathology Division, National Cancer Center Hospital, Tokyo, Japan; 5 Department of Thoracic Oncology, Thoracic Surgery Division, National Cancer Center Hospital, Tokyo, Japan; The University of Arizona, United States of America

## Abstract

The aim of this study was to clarify the significance of DNA methylation alterations at precancerous stages of lung adenocarcinoma. Using single-CpG resolution Infinium array, genome-wide DNA methylation analysis was performed in 36 samples of normal lung tissue obtained from patients without any primary lung tumor, 145 samples of non-cancerous lung tissue (N) obtained from patients with lung adenocarcinomas, and 145 samples of tumorous tissue (T). Stepwise progression of DNA methylation alterations from normal lung tissue to non-cancerous lung tissue obtained from patients with lung adenocarcinomas, and then tumorous tissue samples, was observed at 3,270 CpG sites, suggesting that non-cancerous lung tissue obtained from patients with lung adenocarcinomas was at precancerous stages with DNA methylation alterations. At CpG sites of 2,083 genes, DNA methylation status in samples of non-cancerous lung tissue obtained from patients with lung adenocarcinomas was significantly correlated with recurrence after establishment of lung adenocarcinomas. Among such recurrence-related genes, 28 genes are normally unmethylated (average β-values based on Infinium assay in normal lung tissue samples was less than 0.2) and their DNA hypermethylation at precancerous stages was strengthened during progression to lung adenocarcinomas (Δβ_T–N_>0.1). Among these 28 genes, we focused on 6 for which implications in transcription regulation, apoptosis or cell adhesion had been reported. DNA hypermethylation of the *ADCY5, EVX1, GFRA1, PDE9A,* and *TBX20* genes resulted in reduced mRNA expression in tumorous tissue samples. 5-Aza-2′-deoxycytidine treatment of lung cancer cell lines restored the mRNA expression levels of these 5 genes. Reduced mRNA expression in tumorous tissue samples was significantly correlated with tumor aggressiveness. These data suggest that DNA methylation alterations at precancerous stages determine tumor aggressiveness and outcome through silencing of specific genes.

## Introduction

Lung adenocarcinoma (LADC) is increasingly recognized as a clinicopathologically and molecularly heterogeneous disease: frequent mutations of the *EGFR, KRAS, BRAF, TP53, ERBB2, PIK3CA* and *MET* genes and *EML4-ALK* fusions have been reported in LADCs [1], [2]. In addition, recent whole-exome sequencing has revealed frequent mutation of the *CSMD3* gene [3]. However, the molecular background responsible for the clinicopathological diversity of LADCs is not yet fully understood.

As well as genetic abnormalities, epigenetic changes in human cancers have also been described [4]–[7]. In LADCs, silencing of the *RASSF1A, CDKN2A, RARβ, MGMT, APC, DAPK, FHIT* and *CDH13* genes due to DNA hypermethylation around their promoter regions has been frequently observed [8]. In addition, DNA methylation alterations are known to occur even at the early and precancerous stages of carcinogenesis in various organs [5]–[7], [9]. For example, we have reported that DNA hypermethylation at the D17S5 locus, where the *HIC-1* tumor suppressor gene has been identified, is evident even in non-cancerous lung tissue obtained from patients with non-small cell lung cancers, and is correlated with smoking history [10]. Other researchers have also reported DNA hypermethylation of specific tumor-related genes at precancerous stages associated with cigarette smoking [8], [11]. However, it has been unclear whether DNA methylation status is simply altered at precancerous stages or whether DNA methylation alterations at these stages actually result in gene expression alterations in established LADCs. Moreover, in organs other than the lung, it has been suggested that DNA methylation profiles at precancerous stages may determine tumor aggressiveness and outcome [12]–[14]. However, the clinicopathological impact of DNA methylation alterations at precancerous stages during lung carcinogenesis has not been clarified.

Recently, genome-wide DNA methylation analysis using the single-CpG resolution Infinium array has made it possible to interrogate 27,000 highly informative CpG sites, i.e. an average of two CpG sites within the proximal promoter regions of the transcription start sites of each of 14,475 consensus coding sequences in the National Center for Biotechnology Information Database, especially 3 to 20 CpG sites for more than 200 cancer-related and imprinted genes [15]. Although a few studies of lung cancers employing the Infinium assay have been reported [16], [17], they did not focus on precancerous stages. In order to clarify the significance of DNA methylation alterations at precancerous stages of lung carcinogenesis, we performed the Infinium assay in association with mRNA expression and clinicopathological analyses of 36 samples of normal lung tissue (C) obtained from patients without any primary lung tumors, 145 samples of non-cancerous lung tissue (N) from patients with LADCs, and 145 corresponding samples of tissue from the tumors (T) themselves. Although the molecular classification of LADCs based on the results of the Infinium assay will be published elsewhere, we focused on specific genes methylated at precancerous stages in the present study.

## Materials and Methods

### Patients and Tissue Samples

The 145 paired samples of N and the corresponding T were obtained from patients with primary LADCs who underwent lung resection at the National Cancer Center Hospital, Japan, between December 1997 and March 2008. These patients had undergone complete resection and had not received any preoperative treatment or adjuvant therapy after surgery. Eighty-one patients were males and 64 were females with a median age of 61 years (range, 30–81 yr). Histological diagnosis and grading were based on the 2004 World Health Organization classification [18]. Recurrence was diagnosed by clinicians on the basis of physical examination and imaging modalities such as computed tomography, magnetic resonance imaging, scintigraphy or positron-emission tomography, and sometimes confirmed histopathologically by biopsy.

For comparison, 36 C samples were obtained from materials that had been surgically resected from patients without any primary lung tumor. Twenty-two of these patients were males and 14 were females, with a median age of 63 years (range, 27–83 yr). Thirty-five had undergone lung resection for metastatic lesions of primary cancers of the colon, rectum, kidney, urinary bladder, thyroid, breast, pancreas, ampulla of Vater and salivary gland, osteosarcoma, synovial sarcoma, leiomyosarcoma, rhabdmyosarcoma, liposarcoma, dermatofibrosarcoma, and myxofibrosarcoma. The remaining one patient had undergone chest wall resection for lipoma with removal of adjacent lung tissue. Histological observation confirmed that all of the C samples showed no remarkable histological abnormality and did not contain any contaminating tumor cells that had metastasized from organs other than the lung.

Tissue specimens were provided by the National Cancer Center Biobank, Japan. This study was approved by the Ethics Committee of the National Cancer Center, Tokyo, Japan, and was performed in accordance with the Declaration of Helsinki 1975. All patients included in this study provided written informed consent.

### Cell Lines

The characteristics of the four lung cancer cell lines used in this study are summarized in Table S1.

### Infinium Assay

Genomic DNA was extracted using a QIAamp DNA Mini kit (Qiagen, Valencia, CA, USA) and phenol-chloroform extraction followed by dialysis [19] from all tissue samples and cell lines, respectively. Five-hundred-nanogram aliquots of DNA were subjected to bisulfite conversion using an EZ DNA Methylation-Gold Kit (Zymo Research, Irvine, CA, USA). DNA methylation status at 27,578 CpG loci was examined at single-CpG resolution using the Infinium HumanMethylation27 Bead Array (Illumina, San Diego, CA, USA). After hybridization, the specifically hybridized DNA was fluorescence-labeled by a single-base extension reaction and detected using a BeadScan reader (Illumina) in accordance with the manufacturer’s protocols. The data were then assembled using GenomeStudio methylation software (Illumina). At each CpG site, the ratio of the fluorescence signal was measured using a methylated probe relative to the sum of the methylated and unmethylated probes, i.e. the so-called β-value, which ranges from 0.00 to 1.00, reflecting the methylation level of an individual CpG site.

### Quantitative Real-time Reverse Transcription (RT)-PCR Analysis

Total RNA was extracted from 132 N and 151 T samples for which additional tissue specimens were available and cell lines using TRIzol reagent (Life Technologies, Carlsbad, CA, USA) in accordance with the manufacturer’s instructions. cDNA was synthesized from total RNA with random primers using SuperScript III Reverse Transcriptase (Life Technologies) and pre-amplified using TaqMan PreAmp Master Mix (Life Technologies).

To evaluate mRNA expression levels, fluorescence-labeled locked nucleic acid hydrolysis probes were selected from the Universal Probe Library collection (Roche Applied Science, Mannheim, Germany) and specific PCR primers yielding intron-spanning amplicons were designed using ProbeFinder assay design software (https://www.roche-applied-science.com/sis/rtpcr/upl/index.jsp?id=UP030000). The probe ID and primer sequences are summarized in Table S2. Quantitative real-time PCR was performed using TaqMan Universal Master Mix II (Life Technologies) and the relative standard curve method in the BioMark HD System (Fluidigm, South San Francisco, CA, USA). Ct values were normalized to that of *GAPDH* in the same sample. All assays were performed in triplicate.

### 5-aza-2′-deoxycytidine (5-aza-dC) Treatment

A549, PC9, VMRC-LCD and EBC-1 cells were seeded at a density of 9×10^5^ cells per 15-cm dish on day 0 and then allowed to attach for a 24-h period. Then, 5-aza-dC (Sigma-Aldrich, St. Louis, MO, USA) was added to a final concentration of 1 µM. Cells were passaged at a subculture ratio of 1∶2 on day 3. At 24 h after replating, 5-aza-dC was added again to the same final concentration. Since toxicity had been obvious during preliminary experiments, the final concentration of 5-aza-dC was reduced to 0.1 µM for EBC-1 cells. Genomic DNA and total RNA were extracted from all cells on days 3 and 6.

### Statistics

In the Infinium assay, all CpG sites on chromosomes X and Y were excluded, to avoid any gender-specific methylation bias. The call proportions (*P*-values for detection of signals above the background <0.01) for 31 probes (shown in Table S3) in all of the tissue samples examined were less than 90%. Since such a low proportion may be attributable to polymorphism at the probe CpG sites, these 31 probes were excluded from the present assay, leaving a final total of 26,455 autosomal CpG sites.

Infinium probes showing ordered differences from 36 C to 145 N, and then to the 145 T samples themselves, were examined by the cumulative logit model adjusted by sex, age and experimental batch (*P*<1×10^−14^). Correlations between β-values in N and T samples and recurrence were assessed by the Cox regression model adjusted by sex, age and experimental batch (*P*<0.001). Benjamini-Hochberg correction was performed to adjust for multiple testing. Differences of β-values and mRNA expression levels between N and T samples were examined by Mann-Whitney U test. Correlations between mRNA expression levels and clinicopathological parameters were assayed by analysis of variance between groups (ANOVA) and Welch’s T-test: after adjusted Bonferroni correction to adjust for multiple testing, corrected *P* values of <0.05 were considered to be significant. All statistical analyses were performed using programming language R.

## Results

### DNA Methylation Profiles Associated with Recurrence are Established at Precancerous Stages

DNA methylation levels of CpG sites of the *CNTNAP2*, *EVX1*, *GFRA1*, *PDE9A* and *TBX20* genes based on the Infinium assay were clearly verified using the quantitative pyrosequencing method (Figure S1), indicating the reliability of the Infinium assay. The cumulative logit model (*P*<1×10^−14^) revealed ordered progression of DNA methylation alterations from C to N, and then to T samples, on 3,270 probes; DNA methylation alterations occurred even in N samples compared to C samples, and such DNA methylation alterations were inherited by, or strengthened in, T samples, indicating that Ns were at precancerous stages with DNA methylation alterations. Among the 3,270 probes, the number showing average β-values in T samples minus average β-values in C samples (Δβ_T-C_) of >0.1 and <−0.1 were 1,209 and 1,056, respectively. Thus, when we defined differentially methylated probes as probes showing a Δβ_T-C_ value of >0.1 or <−0.1, the false positivity rate by the cumulative logit model was 4.3%.

**Figure 1:**
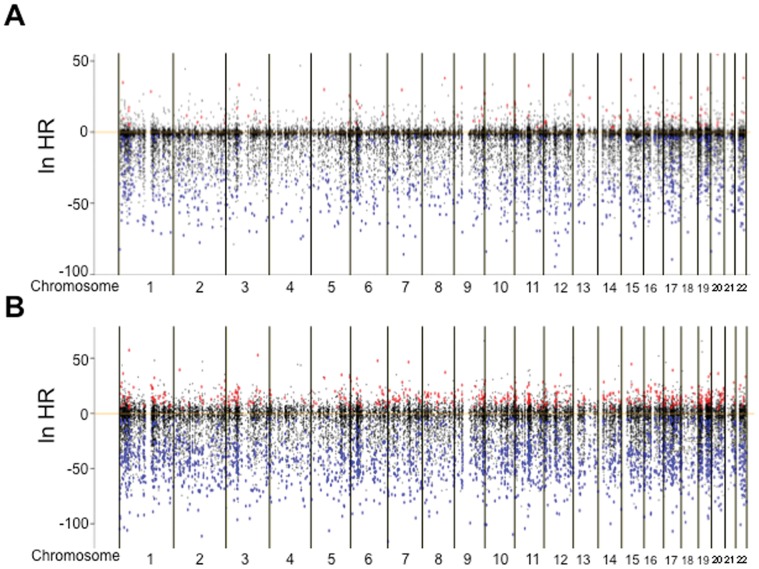
Hazard ratio (HR) obtained from the Cox regression model. Correlation between DNA methylation status (average β-values) and recurrence was examined in 145 samples of tumorous tissue (**A**) and 145 samples of the corresponding non-cancerous lung tissue (**B**) obtained from patients with lung adenocarcinomas who had undergone complete resection and had not received any adjuvant therapy after surgery. All of the examined 26,455 probes of the Infinium array are shown along the chromosomes. Red color means that higher β-values of the probes were observed in recurrence-positive patients than in recurrence-negative patients (*P*<0.001). Blue color means that lower β-values of the probes were observed in recurrence-positive patients than in recurrence-negative patients (*P*<0.001).

Correlations between DNA methylation status and recurrence were examined using the Cox regression model (*P*<0.001) for 145 patients. In T samples, DNA methylation status on 944 probes for the 916 genes was significantly correlated with recurrence: on 87 probes (red dots in Figure 1A), higher β-values were observed in recurrence-positive patients than in recurrence-negative patients, whereas lower β-values on 857 probes (blue dots in Figure 1A) were observed in recurrence-positive patients. Surprisingly, even in N samples, the DNA methylation status on 2,215 probes for the 2,083 genes was significantly correlated with recurrence: on 425 probes (red dots in Figure 1B), higher β-values were observed in recurrence-positive patients than in recurrence-negative patients, whereas lower β-values on 1,790 probes (blue dots in Figure 1B) were observed in recurrence-positive patients.

In order to identify recurrence-related genes that are normally unmethylated and for which DNA hypermethylation at precancerous stages is strengthened in the established LADCs, among the 425 probes (red dots in Figure 1B), we initially focused on 28 probes for which the average β-values in C samples (β_C_) were less than 0.2 and the average β-values in T samples minus that in the corresponding N samples (Δβ_T–N_) were more than 0.1 (Table 1).

**Table 1:** The 28 probes for which the average β-value in N samples (β_N_) was higher in recurrence-positive patients than in recurrence-negative patients, for which the average β-value in C samples (β_C_) was less than 0.2, and for which the average β-value in T samples minus that in corresponding N samples (Δβ_T–N_) was more than 0.1.

Target ID^a^	Chromosome	Position^b^	Gene symbol	*P* c	Adjusted *P* d
cg00516481	21	44,073,202	*PDE9A*	9.996×10^−4^	1.193×10^−2^
cg01295203	8	70,984,199	*PRDM14*	8.156×10^−5^	3.236×10^−3^
cg02008154	7	35,293,537	*TBX20*	7.188×10^−4^	9.917×10^−3^
cg02909790	6	26,271,587	*HIST1H3G*	2.896×10^−6^	5.787×10^−4^
cg03538436	12	117,799,370	*NOS1*	7.227×10^−4^	9.944×10^−3^
cg03963198	5	1,882,871	*IRX4*	5.133×10^−4^	8.186×10^−3^
cg06005396	19	590,541	*HCN2*	9.485×10^−4^	1.158×10^−2^
cg06269753	8	72,755,871	*MSC*	8.627×10^−7^	3.426×10^−4^
cg07651242	7	45,614,720	*ADCY1*	7.403×10^−4^	1.010×10^−2^
cg11612345	6	168,842,491	*SMOC2*	4.264×10^−6^	6.753×10^−4^
cg12087643	10	118,033,370	*GFRA1*	3.144×10^−9^	2.489×10^−5^
cg12265829	14	24,804,022	*ADCY4*	7.265×10^−4^	9.984×10^−3^
cg13262687	4	147,559,579	*POU4F2*	6.246×10^−5^	2.795×10^−3^
cg13449778	1	179,712,298	*FAM163A*	8.195×10^−7^	3.426×10^−4^
cg13878010	3	123,167,276	*ADCY5*	2.339×10^−6^	5.220×10^−4^
cg16254309	7	145,814,152	*CNTNAP2*	2.917×10^−4^	6.240×10^−3^
cg16387606	1	149,804,293	*HIST2H4A*	7.441×10^−4^	1.011×10^−2^
cg16604516	3	13,590,419	*FBLN2*	7.181×10^−4^	9.916×10^−3^
cg16652259	2	172,949,501	*DLX1*	4.175×10^−4^	7.386×10^−3^
cg17191178	3	157,824,217	*SHOX2*	8.872×10^−5^	3.353×10^−3^
cg18454685	17	48,639,239	*CACNA1G*	4.326×10^−4^	7.487×10^−3^
cg20286200	6	133,562,267	*EYA4*	1.541×10^−9^	2.038×10^−5^
cg21087137	12	75,728,469	*GLIPR1L1*	1.965×10^−6^	4.821×10^−4^
cg22461835	8	26,723,365	*ADRA1A*	7.297×10^−4^	9.998×10^−3^
cg23418591	20	57,090,317	*LOC149773*	4.075×10^−4^	7.287×10^−3^
cg25302419	5	11,904,015	*CTNND2*	5.149×10^−4^	8.194×10^−3^
cg25764191	10	105,037,215	*INA*	3.834×10^−4^	7.026×10^−3^
cg27626299	7	27,282,431	*EVX1*	2.164×10^−4^	5.423×10^−3^

a Probe ID for the Infinium HumanMethylation27 Bead Array (Illumina).

b National Center for Biotechnology Information database (Genome Build 37).

c Non-adjusted *P*-values and.

d Benjamini-Hochberg-adjusted *P*-values for the Cox regression model used for evaluation of correlation with recurrence.

### Silencing of Recurrence-related Genes due to DNA Hypermethylation

**Figure 2:**
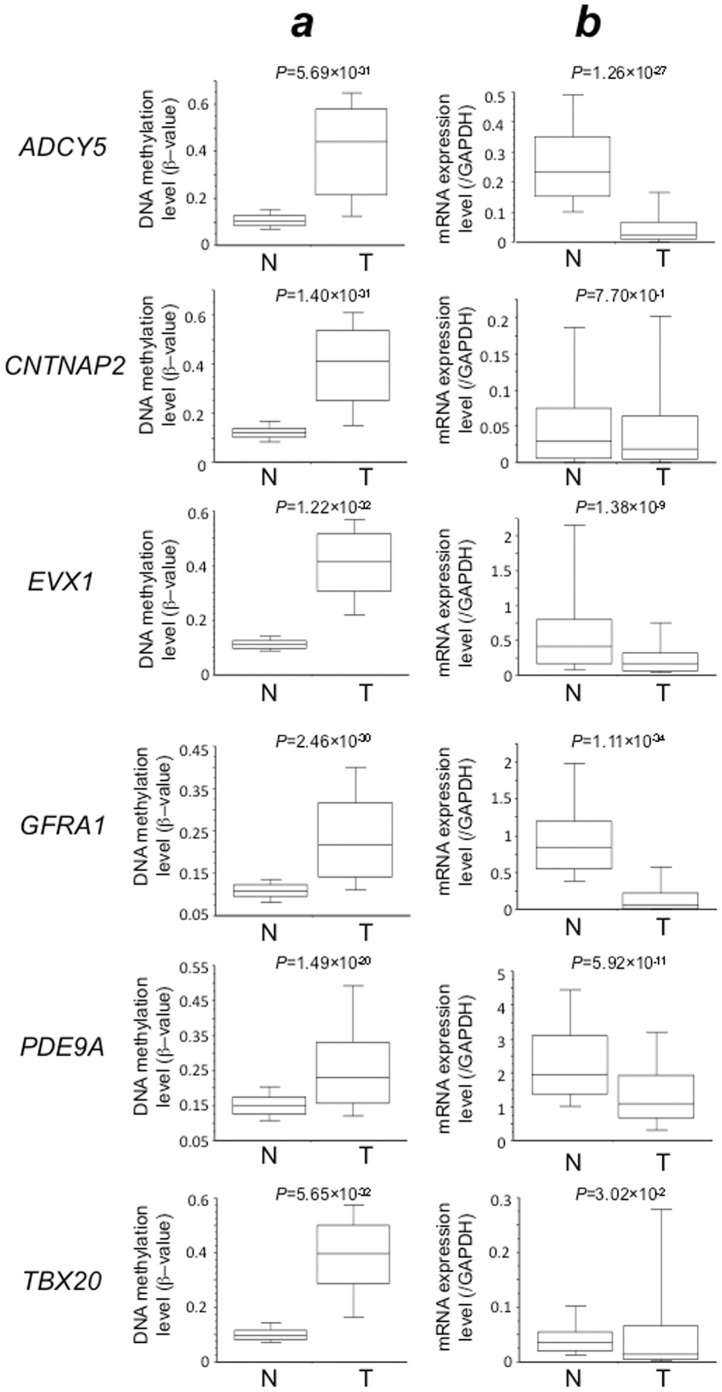
Correlation between DNA methylation levels and mRNA expression levels. DNA methylation levels (average β-values) (**A**) and mRNA expression levels (**B**) for the *ADCY5, CNTNAP, EVX1, GFRA1, PDE9A* and *TBX20* genes in samples of non-cancerous lung tissue (N) from patients with lung adenocarcinomas and samples of the corresponding tumorous tissue (T) were determined by Infinium assay and quantitative real-time reverse transcription-PCR analysis, respectively. DNA methylation levels for all six genes were significantly higher in T samples than in N samples, and levels of expression of mRNAs for the *ADCY5, EVX1, GFRA1, PDE9A* and *TBX20* genes were significantly lower in T samples than in N samples, although the reduction in the expression of the *CNTNAP2* gene did not reach statistical significance. These results suggested that DNA hypermethylation of the *ADCY5, EVX1, GFRA1, PDE9A* and *TBX20* genes may result in reduced mRNA expression in tissue samples from the same cohort.

Among the 28 genes listed in Table 1, we further focused on 6 genes (*ADCY5* [20], [21], *CNTNAP2* [22], [23], *EVX1* [24], *GFRA1* [25], *PDE9A* [26], [27] and *TBX20* [28]) for which implications in transcription regulation, apoptosis or cell adhesion had been reported. Quantitative real-time RT-PCR analysis of these 6 genes was performed in 132 N and 151 T samples for which total RNA was available. mRNA expression levels for the *ADCY5, EVX1, GFRA1, PDE9A* and *TBX20* genes in T samples were significantly lower than those in N samples, although the reduced expression of the *CNTNAP2* gene did not reach statistical significance (Figure 2B). The DNA methylation statuses (β-values in N and T samples) of the 6 genes are also shown in Figure 2A; the data suggested that DNA hypermethylation of these genes might result in reduction of mRNA expression in tissue samples from the same cohort.

**Figure 3:**
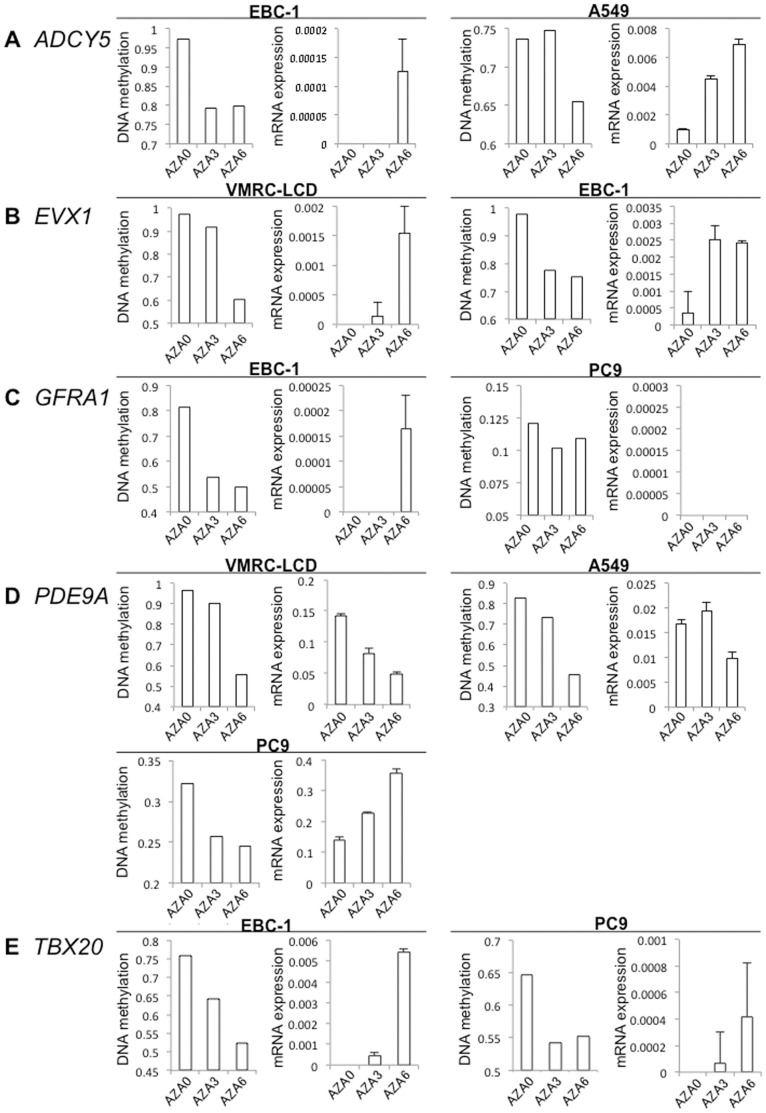
DNA methylation levels and mRNA expression levels after 5-aza-2′-deoxycytidine (5-aza-dC) treatment. DNA methylation levels (β-values) and mRNA expression levels for the *ADCY5* (**A**), *EVX1* (**B**), *GFRA1* (**C**), *PDE9A* (**D**) and *TBX20* (**E**) genes were determined by Infinium assay and quantitative real-time reverse transcription-PCR analysis, respectively. The error bars represent the standard deviation for triplicate quantitative real-time RT-PCR analyses. DNA methylation levels and mRNA expression levels on days 3 (AZA3) and 6 (AZA6) were compared with those of untreated cells (AZA0). After 5-aza-dC treatment, reduction of DNA methylation levels and restoration of the mRNA expression levels of *ADCY5* (**A**), *EVX1* (**B**) and *TBX20* (**E**) were observed in both of the cell lines used. In panel **C**, since reduction of the DNA methylation level was not induced by 5-aza-dC in PC9 cells, restoration of mRNA expression did not occur in these cells. Panel **D** shows reduction of the DNA methylation level and restoration of the mRNA expression level in PC9 cells.

DNA methylation levels of the *ADCY5, EVX1, GFRA1, PDE9A* and *TBX20* genes in lung cancer cell lines A549, PC9, VMRC-LCD and EBC-1 are shown in Figure S2. To examine the effects of the DNA methylation inhibitor, the top two cell lines showing the highest DNA methylation levels (β-values) were selected for each gene. In fact, mRNA expression levels determined by quantitative real-time RT-PCR analysis of the genes (with the exception of PDE9A) were extremely low in the cell lines selected. 5-aza-dC treatment induced marked reduction of DNA methylation levels and restored the mRNA expression levels of *ADCY5, EVX1, GFRA1* and *TBX20* (Figure 3). With regard to *GFRA1*, since reduction of the DNA methylation level was not induced by 5-aza-dC, restoration of mRNA expression did not occur in PC9 cells. Taken together with Figures 2 and 3, the data suggested that the examined genes were silenced due to DNA hypermethylation in the lung cancers. With regard to *PDE9A*, for which the mRNA expression levels were high even in the top two cell lines showing the highest levels of DNA methylation, 5-aza-dC treatment did not further increase the mRNA expression level in the two cell lines. The PC9 cells were then additionally treated with 5-aza-dC, and restoration of *PDE9A* mRNA expression due to DNA demethylation was confirmed in the cells (Figure 3).

### Clinicopathological Impact of Reduced Expression of mRNA for Recurrence-related Genes

Reduced expression of mRNA for *ADCY5, EVX1, GFRA1* and *PDE9A* in T samples was correlated with clinicopathological parameters reflecting tumor aggressiveness, such as a larger tumor diameter, higher histological grade, blood vessel invasion, pleural invasion and tumor anthracosis (Table 2), although their mRNA expression levels were not predictors of recurrence that were independent of known parameters such as pathological-TNM stage and lymph node metastasis (Table S4). With regard to the correlation with histological subtype, levels of mRNA expression for *GFRA1* and *PDE9A* were significantly higher in lepidic-type LADCs showing a less invasive growth pattern than in other histological subtypes.

**Table 2:** Correlation between mRNA expression levels of recurrence-related genes and clinicopathological factors.

Clinicopathological parameters	Number of tumors	*ADCY5*		*EVX1*		*GFRA1*		*PDE9A*		*TBX20*	
		Expression^a^	*P* ^b^	Expression^a^	*P* ^b^	Expression^a^	*P* ^b^	Expression^a^	*P* ^b^	Expression^a^	*P* ^b^
Tumor diameter											
<2.5 cm	43	−4.95±2.10	1.63×10^−1c^	−2.80±1.82	4.24×10^−1c^	−3.27±2.67	1.60×10 ^−3^c	0.50±1.39	3.40×10 ^−2^c	−5.73±2.68	7.65×10^−1c^
≥2.5 cm, <4 cm	61	−5.25±2.39		−2.36±2.16		−4.02±2.86		0.28±1.76		−5.76±2.78	
≥4 cm	47	−6.14±2.76		−3.04±2.02		−5.74±3.28		−0.50±1.59		−6.10±2.84	
Histological subtype^d^											
Lepidic	12	−4.19±1.61	2.10×10^−1c^	−2.73±2.14	1.79×10^−1c^	−1.64±2.35	1.71×10 ^−4^c	0.93±1.07	1.73×10 ^−3^c	−6.24±1.79	1.28×10^−1c^
Acinar	24	−6.03±2.01		−2.91±2.09		−5.41±2.72		−0.20±1.36		−5.77±2.57	
Papillary	69	−5.30±2.35		−2.29±1.99		−3.90±2.65		0.23±1.63		−5.28±2.80	
Micropapillary	15	−4.69±2.19		−2.35±2.10		−3.47±2.87		1.20±1.27		−6.89±1.79	
Solid	28	−6.34±3.24		−3.62±1.88		−6.27±3.62		−0.91±1.81		−6.37±3.11	
Invasive mucinous	3	−4.64±1.46		−3.19±1.24		−2.95±1.43		0.16±1.68		−8.21±0.14	
Histological grades											
G1	56	−4.60±1.80	1.28×10 ^−3^c	−2.68±1.90	4.57×10 ^−2^c	−2.62±1.93	4.80×10 ^−10^	0.55±1.41	1.32×10 ^−4^	−5.48±2.69	9.50×10^−2^
G2	65	−5.58±2.33		−2.32±2.10		−4.59±2.71		0.25±1.57		−5.74±2.67	
G3	30	−6.73±3.20		−3.54±1.92		−7.01±3.60		−1.06±1.77		−6.81±2.94	
Lymphatic invasion											
Negative	52	−5.31±2.43	1.00^e^	−2.76±1.71	1.00^e^	−3.95±3.38	1.00^e^	0.06±1.64	1.00^e^	−5.86±2.42	9.82×10^−1e^
Positive	99	−5.52±2.50		−2.66±2.19		−4.55±2.92		0.12±1.67		−5.85±2.93	
Blood vessel invasion											
Negative	43	−4.89±2.40	2.38×10^−1e^	−2.77±1.84	7.68×10^−1e^	−3.13±3.45	2.81×10 ^−2e^	0.63±1.68	6.39×10^−2e^	−5.71±2.60	1.00^e^
Positive	108	−5.66±2.47		−2.67±2.11		−4.82±2.81		−0.11±1.60		−5.92±2.83	
Pleural invasion											
Negative	78	−5.16±2.42	4.45×10^−1c^	−2.77±2.15	4.60×10^−1c^	−3.65±2.83	7.28×10 ^−3^c	0.28±1.61	5.82×10^−1c^	−5.95±2.51	5.38×10^−1c^
Invasion to the visceral pleura beyond the elastic fiber	33	−5.88±2.68		−2.62±2.07		−4.99±3.54		0.03±1.93		−6.41±2.96	
Invasion to the surface of the visceral pleura	22	−4.96±2.07		−2.16±1.62		−4.01±2.66		0.18±1.29		−4.98±2.82	
Invasion to the parietal pleura	18	−6.47±2.49		−3.15±1.90		−6.56±2.71		−0.65±1.58		−5.52±3.22	
Tumor anthracosis^ f^											
Negative	68	−4.97±2.57	8.27×10^−2e^	−2.37±2.22	2.04×10^−1e^	−3.32±2.80	1.08×10 ^−3^e	0.70±1.46	1.66×10 ^−4^e	−5.51±2.78	1.99×10^−1e^
Positive	82	−5.87±2.30		−2.91±1.79		−5.10±3.02		−0.39±1.66		−6.09±2.69	

a Average of log2-transformed mRNA expression levels/GAPDH ± standard deviation. ^b^Adjusted *P*-values using adjusted Bonferroni correction. ^c^Analysis of variance between groups. ^d^Predominant histological subtypes of LADCs were diagnosed according to the classification devised by the International Association for the Study of Lung Cancer, the American Thoracic Society and the European Respiratory Society [29] ^. e^Welch’s T-test. ^f^Coal dust is accumulated in active fibroblast proliferation foci, which is associated with poorer prognosis of lung adenocarcinoma patients and reflects an active cancer-stromal interaction [30]. *P* values of <0.05 are underlined.

## Discussion

The ‘field cancerization’ phenomenon in the lung has become evident, being especially associated with cigarette smoking [31]. We and other groups have reported DNA methylation of specific genes or chromosomal loci in non-cancerous lung tissue obtained from lung cancer patients, or in lung tissue from cancer-free smokers [8]–[10]. These previous data drew our attention to DNA methylation alterations at precancerous stages of LADC. However, the impact of DNA methylation alterations at precancerous stages on the expression of specific gene and clinicopathological parameters of established cancers has remained unclear. Moreover, previous examinations focusing on precancerous stages in the lung have not involved a genome-wide approach. Although Selamat et al. and Lockwood et al. have reported Infinium assay results for 59 and 43 lung cancer samples, respectively [16], [17], they did not focus on precancerous stages.

Here we have reported the results of the Infinium assay for 326 lung tissue samples including 145 N samples. Our cumulative logit model analysis revealed stepwise progression of DNA methylation alterations from C to N, and then T samples on 3,270 probes. Genome-wide analysis at single-CpG resolution confirmed that DNA methylation alterations actually occurred even at precancerous stages, although the possibility that such alterations observed in N samples had been influenced by differences in tissue composition between C and N samples cannot be completely excluded. Moreover, it was clearly shown that such DNA methylation alterations had clinicopathological impact, since many probes in N samples were significantly correlated with recurrence after establishment of LADCs (Figure 1B). DNA methylation profiles determining outcome are already established at precancerous stages. The finding that the number of probes showing DNA methylation alterations significantly associated with recurrence in N samples was larger than that in T samples may have been due to the fact that passenger DNA methylation alterations occurring during progression from the precancerous stages to established cancers may have masked any clinicopathologically significant DNA methylation profiles in T samples.

Next, we focused on 28 recurrence-related genes that are normally unmethylated and for which DNA hypermethylation in N samples was strengthened in T samples (Table 1). Among these 28 genes, we further focused on *ADCY5, CNTNAP2, EVX1, GFRA1, PDE9A* and *TBX20*, based on their previously reported implications in transcription regulation, apoptosis or cell adhesion. (a) The data in *adcy5*-knockout mice indicated that ADCY5 promotes apoptosis in cardiomyocytes [20], [21]. Although large-scale screening studies of leukemias have identified *ADCY5* as one of the genes that are methylated in leukemic cells [32], [33], the clinicopathological impact of DNA methylation of the *ADCY5* gene has not yet been clarified in human malignancies. (b) CNTNAP2, a glial adhesion molecule, binds extracellularly to contactin 2, an immunoglobulin superfamily neural recognition protein [22]. *CNTNAP2* is known to be a tumor-suppressor gene for gliomas, and is disrupted by chromosomal translocations and gene mutations [23]. Although a large-scale screening study of pancreatic cancers has identified *CNTNAP2* as one of the genes that are methylated in cancer cells [34], the clinicopathological impact of DNA methylation of the *CNTNAP2* gene has not yet been elucidated in human malignancies. (c) *EVX1*, encoding a homeobox protein, functions as a potent repressor of gene transcription and plays an important role during mouse embryogenesis [24]. Although Truong et al. have recently suggested that DNA hypermethylation of the *EVX1* gene may be a predictor of recurrence of prostatic cancers [35], the implications of *EVX1* in human cancers other than prostatic cancer have been unclear. (d) GFRA1 is a receptor for glial cell-derived neurotrophic factor (GDNF) and enriched in the pre- and post-synaptic compartments. GDNF triggers trans-homophilic binding between GFRA1 molecules, resulting in adhesion between *GFRA1*-expressing cells [25]. Overexpression of *GFRA1* has been reported in chemotherapy-sensitive oligodendrogliomas [36]. Although Salamat et al. described *GFRA1* as one of the genes differentially methylated between cancerous and non-cancerous tissue obtained from lung cancer patients subclustered on the basis of DNA methylation profiles [16], the clinicopathological impact of DNA methylation of the *GFRA1* gene was not examined in LADCs. (e) *PDE9A* encodes cGMP-specific phosphodiesterase [26]. PDE9A inhibitor has been reported to induce apoptosis of breast cancer cell lines through caspase 3 activation [27]. On the other hand, breakpoints within the *PDE9A* gene have been frequently observed in B-cell precursor acute lymphoblastic leukemia [36]. However, the implications of DNA methylation in the regulation of *PDE9A* have never been reported in human malignancies or other diseases. (f) TBX20, a member of the T-box transcription factors, is a critical regulator of heart development, and mutations of the human *TBX20* gene result in cardiac malformations [28]. However, the implications of DNA methylation in *TBX20* regulation and of *TBX20* dysfunction in human malignancies have never been reported.

Among these 6 genes, DNA hypermethylation of the *ADCY5, EVX1, GFRA1, PDE9A* and *TBX20* genes was associated with reduced mRNA expression in tissue samples of the same cohort. 5-aza-dC treatment of human lung cancer cell lines restored the expression of the 5 genes, indicating that genes showing DNA hypermethylation at precancerous stages are actually silenced due to DNA hypermethylation during lung carcinogenesis. With regard to *PDE9A*, the level of mRNA expression was not necessarily low and was not always restored by 5-aza-dC treatment in any of the cell lines examined, indicating that DNA methylation is not the only mechanism responsible for *PDE9A* regulation in lung cancers. In addition, there are gaps between the timing of the reduction of DNA methylation and recovery of mRNA expression in *ADCY5* in EBC-1 cells and A549 cells and *GFRA1* in EBC-1 cells, again indicating the possibility that there are alternative mechanisms regulating the mRNA expression levels of these genes other than DNA methylation. Reduced expression of the mRNAs for the *ADCY5, EVX1, GFRA1* and *PDE9A* genes was significantly correlated with clinicopathological parameters (Table 2), indicating that DNA methylation alterations, even from the precancerous stage, ultimately determine the tumor phenotype through gene silencing.

Unlike the present study, which was conducted to clarify the significance of DNA methylation alterations at precancerous stages, many previous studies attempted to establish prognostic factors based on DNA methylation status using candidate gene approaches [37]–[41]. Microarray studies are generally considered to be useful for establishing prognostic biomarkers. In lung cancers, array-based DNA methylation screening [42] has been performed for prognostication. Next-generation sequencing associated with bioinformatics analysis [43] has also been reported for lung cancers. Such previous studies identified the *RASSF1A* [37], *PITX2* [38], *SHOX2* [38], *TFPI-2* [39], *FHIT* [40], *p16* [41], *CDH13* [41], and *APC* [41] genes as predictors of recurrence of lung cancers. The genes for which DNA methylation status at precancerous stages (β_N_ values) was associated with recurrence in the present study are different from the prognostic biomarkers selected in previous studies based on the DNA methylation status of tumorous tissues themselves.

In summary, DNA methylation status is not simply altered at precancerous stages, and is significantly correlated with recurrence after establishment of LADCs. DNA methylation alterations at precancerous stages are strengthened in comparison to normal lung tissues during progression to established LADC. DNA methylation profiles at precancerous stages may determine tumor aggressiveness through alterations in the expression of mRNAs for specific genes.

## Supporting Information

Figure S1 **Verification of the results of the Infinium assay using pyrosequencing methods.** To overcome the PCR bias in pyrosequencing, the PCR conditions were optimized for each primer set, as described previously (Nagashio R, et al. Int J Cancer 129∶1170, 2011). Pyrosequencing was performed for the *CNTNAP2, EVX1*, *GFRA1, PDE9A* and *TBX20* genes using 9 representative T samples and 9 corresponding N samples. β-values obtained from the Infinium assay were strongly correlated with DNA methylation levels obtained by pyrosequencing in all 5 genes, indicating that the results of the Infinium assay were successfully verified.
(TIF)

Figure S2 **DNA methylation levels for the** ***ADCY5, EVX1, GFRA1, PDE9A*** **and** ***TBX20*** **genes in lung cancer cell lines.** DNA methylation levels (β-values) for the *ADCY5, EVX1, GFRA1, PDE9A* and *TBX20* genes in all 4 of the lung cancer cell lines were examined by Infinium assay. To examine the effects of the DNA methylation inhibitor, 5-aza-2′-deoxycytidine, the top two cell lines showing the highest DNA methylation levels were selected for each gene: EBC-1 and A549 cells for the *ADCY5* gene, VMRC-LCD and EBC-1 cells for the *EVX1* gene, EBC-1 and PC9 cells for the *GFRA1* gene, VMRC-LCD and A549 cells for the *PDE9A* gene, and EBC-1 and PC9 cells for the *TBX20* gene.
(TIF)

Table S1 **Characteristics of the lung cancer cell lines.**
(PDF)

Table S2 **Probe ID and primer sequences for quantitative real-time reverse transcription-PCR.**
(PDF)

Table S3 **The probes for which call proportions in all examined tissue samples were less than 90%.**
(PDF)

Table S4 **Multivariate analysis of clinicopathological parameters and mRNA expression levels of selected genes associated with recurrence in patients with lung adenocarcinomas.**
(PDF)
