# Supplementary figures and images for: DNA Methylation Profiles at Precancerous Stages Associated with Recurrence of Lung Adenocarcinoma
Source: PLoS One. 2013 Mar 27;8(3):e59444. doi: 10.1371/journal.pone.0059444 (PMC3609833; doi:10.1371/journal.pone.0059444)

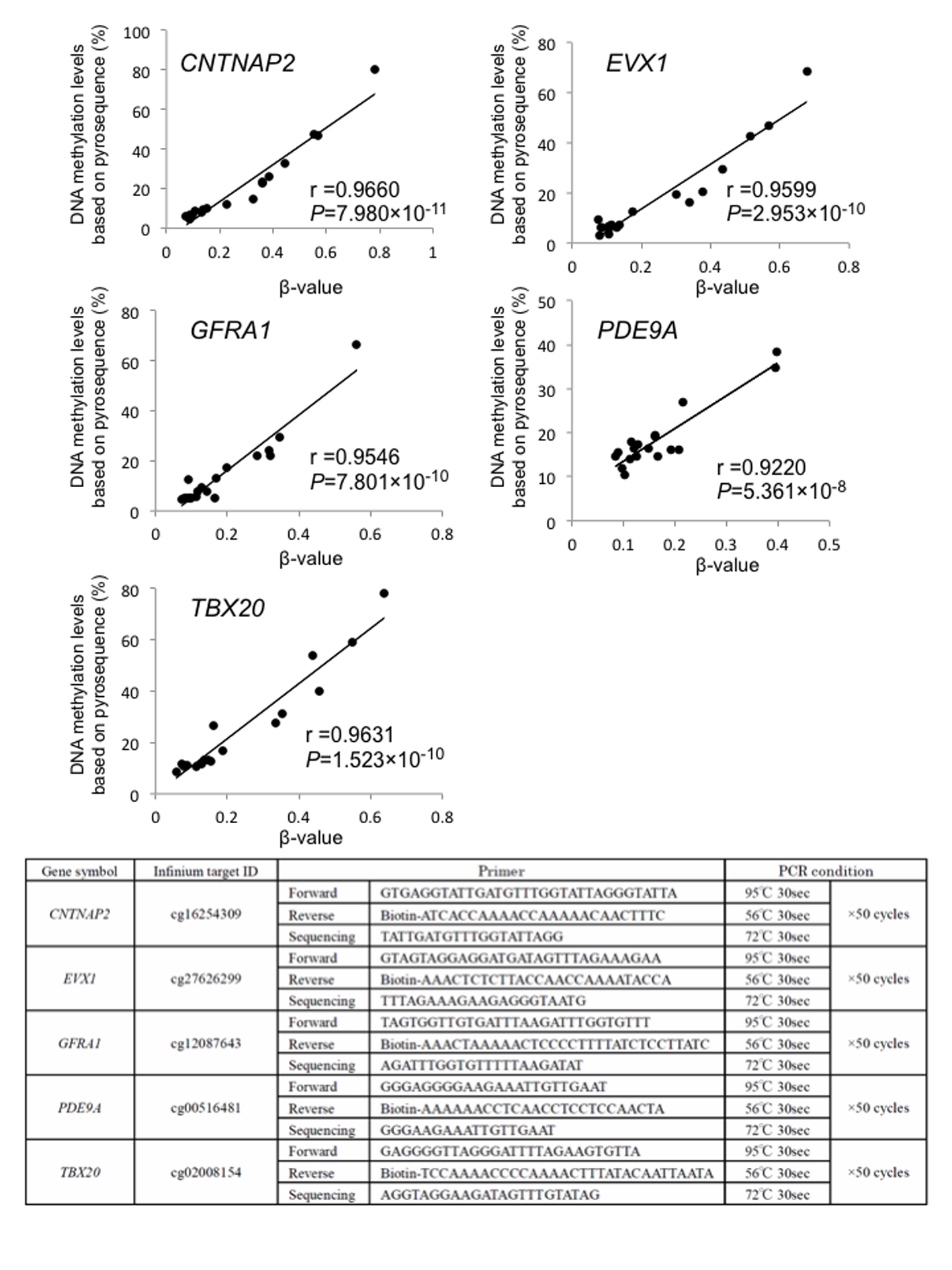

Supplement: Figure S1 — Verification of the results of the Infinium assay using pyrosequencing methods. To overcome the PCR bias in pyrosequencing, the PCR conditions were optimized for each primer set, as described previously (Nagashio R, et al. Int J Cancer 129∶1170, 2011). Pyrosequencing was performed for the CNTNAP2, EVX1, GFRA1, PDE9A and TBX20 genes using 9 representative T samples and 9 corresponding N samples. β-values obtained from the Infinium assay were strongly correlated with DNA methylation levels obtained by pyrosequencing in all 5 genes, indicating that the results of the Infinium assay were successfully verified. (TIF) [file pone.0059444.s001.tif]

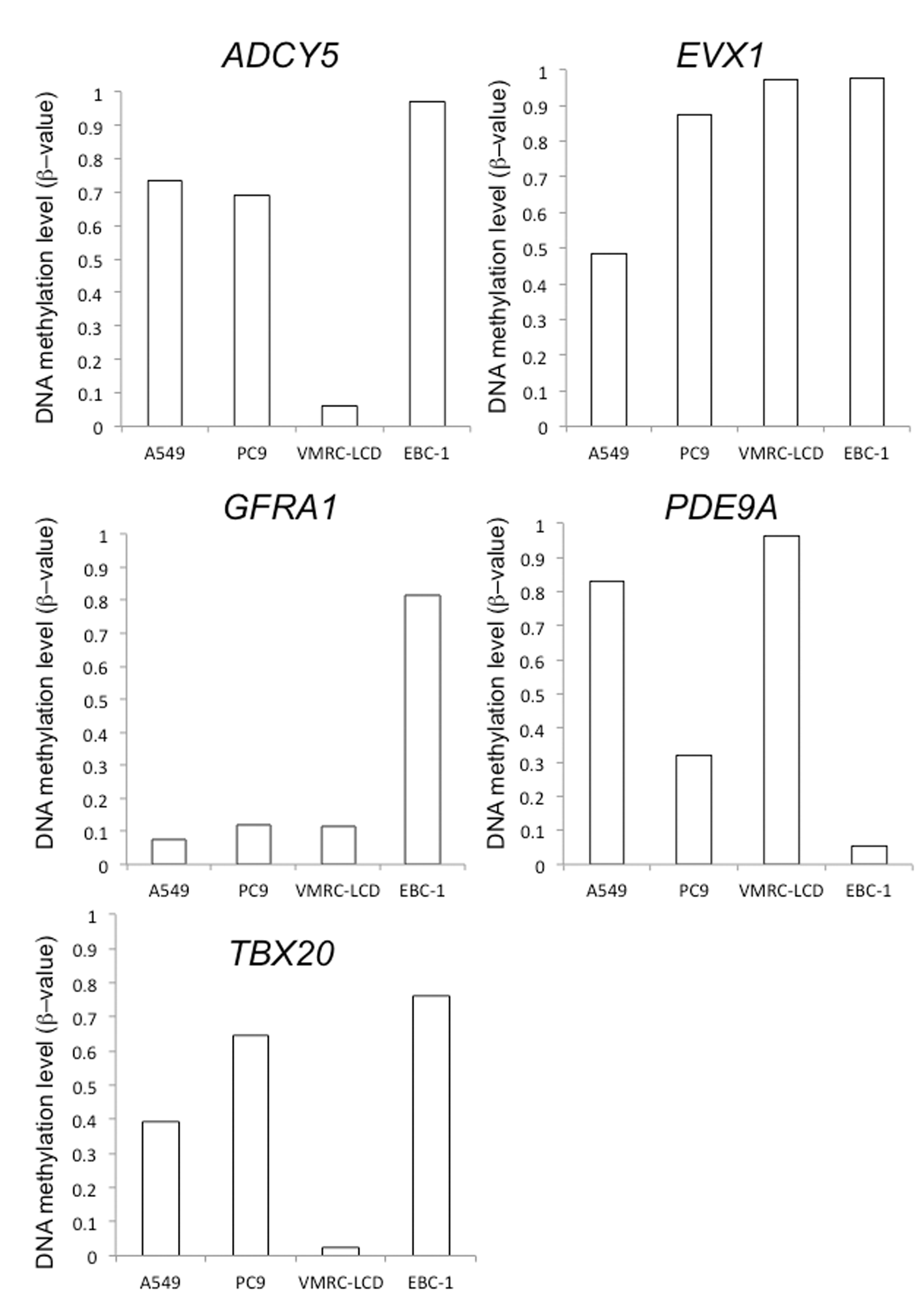

Supplement: Figure S2 — DNA methylation levels for the ADCY5, EVX1, GFRA1, PDE9A and TBX20 genes in lung cancer cell lines. DNA methylation levels (β-values) for the ADCY5, EVX1, GFRA1, PDE9A and TBX20 genes in all 4 of the lung cancer cell lines were examined by Infinium assay. To examine the effects of the DNA methylation inhibitor, 5-aza-2′-deoxycytidine, the top two cell lines showing the highest DNA methylation levels were selected for each gene: EBC-1 and A549 cells for the ADCY5 gene, VMRC-LCD and EBC-1 cells for the EVX1 gene, EBC-1 and PC9 cells for the GFRA1 gene, VMRC-LCD and A549 cells for the PDE9A gene, and EBC-1 and PC9 cells for the TBX20 gene. (TIF) [file pone.0059444.s002.tif]
