# Supplementary material for: DNA Methylation Profiles at Precancerous Stages Associated with Recurrence of Lung Adenocarcinoma
Source: PLoS One. 2013 Mar 27;8(3):e59444. doi: 10.1371/journal.pone.0059444 (PMC3609833; doi:10.1371/journal.pone.0059444)
Supplement: Table S1 — Characteristics of the lung cancer cell lines. (PDF) [file pone.0059444.s003.pdf]

Table S1. Characteristics of the lung cancer cell lines.

Cell line	Histology	Reference	Supplier
A549	Adenocarcinoma	*J Nat Cancer Inst* 1973;**51**:1417-23.	American Type Culture Collection (Manassas, VA, USA)
PC9	Adenocarcinoma	*Br J Cancer* 1979;**39**:15-23.	Immuno-Biological Laboratories (Gunma, Japan)
VMRC-LCD	Adenocarcinoma	*Clin Exp Metastasis* 1991;**9**:517-27.	Immuno-Biological Laboratories (Gunma, Japan)
EBC-1	Squamous cell carcinoma	*Br J Cancer* 1989;**59**:761-5.	Health Science Research Resources Bank (Osaka, Japan)
