# Supplementary material for: DNA Methylation Profiles at Precancerous Stages Associated with Recurrence of Lung Adenocarcinoma
Source: PLoS One. 2013 Mar 27;8(3):e59444. doi: 10.1371/journal.pone.0059444 (PMC3609833; doi:10.1371/journal.pone.0059444)
Supplement: Table S2 — Probe ID and primer sequences for quantitative real-time reverse transcription-PCR. (PDF) [file pone.0059444.s004.pdf]

Table S2. Probe ID and primer sequences for quantitative real-time reverse transcription-PCR.

Gene	Accession Number[a]	Probe ID[b]	Primer sequences
ADCY5	NM_183357.2	#64	Forward 5'-TCCAGAAACATGACAACGTGA-3'<br>Reverse 5'-TGACCAGTTCCTGTGCAGTG-3'
CNTNAP2	NM_014141.5	#59	Forward 5'-CCAAATCGATATTTCCTCAGGT-3'<br>Reverse 5'-CTTGGCTAGGAAGCGAACC-3'
EVX1	NM_001989.3	#19	Forward 5'-TTCACCCGAGAGCAGATTG-3'<br>Reverse 5'-CACACCTTGATGGTGGTTTC-3'
GFRA1	NM_005264.4	#20	Forward 5'-GGACTCCTGCAAGACGAATTA-3'<br>Reverse 5'-CAGCTGCTGACAGACCTTGA-3'
PDE9A	NM_002606.2	#22	Forward 5'-CCTAGAGAAACGCGTGGAAT-3'<br>Reverse 5'-TCTTCTTAATGTCACTCTTGCATTTC-3'
TBX20	NM_020417.1	#51	Forward 5'-GGGACAAATTCCATGAGCTG-3'<br>Reverse 5'-ACCCGGATGGTTGGAAAC-3'
GAPDH	NM_002046.4	#60	Forward 5'-AGCCACATCGCTCAGACAC-3'<br>Reverse 5'-GCCCAATACGACCAAATCC-3'

[a]GenBank (National Center for Biotechnology Information). [b]Human Universal ProbeLibrary (Roche Applied Science).
