# Supplementary material for: DNA Methylation Profiles at Precancerous Stages Associated with Recurrence of Lung Adenocarcinoma
Source: PLoS One. 2013 Mar 27;8(3):e59444. doi: 10.1371/journal.pone.0059444 (PMC3609833; doi:10.1371/journal.pone.0059444)
Supplement: Table S3 — The probes for which call proportions in all examined tissue samples were less than 90%. (PDF) [file pone.0059444.s005.pdf]

Table S3. The probes for which call proportions in all examined tissue samples were less than 90%.

Target ID [a]	Chromosome	Gene symbol	Call proportion (%) [b]
cg03727165	8	C8orf4	19.1
cg13733733	19	LILRA3	42.8
cg15307449	17	C17orf85	51.0
cg17901463	1	GSTM1	52.4
cg19481811	4	UGT2B17	54.6
cg00729708	15	LASS3	61.6
cg24330042	22	GSTT1	67.6
cg20945531	6	FLJ34503	67.9
cg11680741	17	MYH8	68.8
cg13798376	12	EPYC/DSPG3	69.6
cg19584733	9	DENND4C	69.8
cg14740251	19	SIGLEC5	71.0
cg12466095	14	ATG2B	72.5
cg21162961	4	RRH	74.9
cg02039053	4	ADH1A	75.1
cg20630386	4	AFP	75.4
cg24346429	20	GNAS	76.6
cg27356438	7	NPVF	76.6
cg06318853	4	INPP4B	77.3
cg08634464	19	ZNF57	79.2
cg27257987	19	PSG4	80.7
cg02655623	17	C17orf85	80.9
cg02549424	22	AIFM3	81.6
cg26415655	5	IL31RA	83.3
cg15309578	2	PELI1	84.5
cg19507591	11	ZFPL1	85.3
cg03536003	4	TMPRSS11D	85.7
cg12014368	21	MRAP	86.0
cg00706536	17	ATP6V0A1	87.2
cg19831575	11	FGF4	87.2
cg09224952	3	KCTD6	88.2
cg17687883	2	MTHFD2	88.4
cg12255284	8	C8orf45	88.9
cg18236721	9	IL33	88.9
cg01607495	8	BAG4	89.1
cg14021698	21	KRTAP21-2	89.1
cg21168622	19	ZNF350	89.1
cg10978346	4	INPP4B	89.6
cg24272907	12	RIMBP2	89.6
cg24856383	3	SIAH2	89.6

[a]Probe ID for the Infinium HumanMethylation27 Bead Array (Illumina). [b] *P*-values for detection of signal above the background <0.01.

Since low call proportions may be attributable to polymorphism at the probe CpG sites, the above 40 probes were excluded from the present assay.
