# Supplementary material for: DNA Methylation Profiles at Precancerous Stages Associated with Recurrence of Lung Adenocarcinoma
Source: PLoS One. 2013 Mar 27;8(3):e59444. doi: 10.1371/journal.pone.0059444 (PMC3609833; doi:10.1371/journal.pone.0059444)
Supplement: Table S4 — Multivariate analysis of clinicopathological parameters and mRNA expression levels of selected genes associated with recurrence in patients with lung adenocarcinomas. (PDF) [file pone.0059444.s006.pdf]

Table S4. Multivariate analysis of clinicopathological parameters and mRNA expression levels of selected gene associated with recurrence in patients with lung adenocarcinomas

(a) Clinicopathological parameters and mRNA expression levels of ADCY5

	Coefficient (Standard error)	*Z*	*P*
Pathological TNM stage[a]	0.10030 (0.08912)	1.126	0.260746
Lymph node metastasis[b]	0.62993 (0.17095)	3.685	0.000226
mRNA expression levels of ADCY5	0.10936 (0.07209)	1.517	0.129289

[a]Pathological Tumor-Node-Metastasis stage. [b]Positive or negative.

(b) Clinicopathological parameters and mRNA expression levels of EVX1

	Coefficient (Standard error)	*Z*	*P*
Pathological TNM stage[a]	0.099932 (0.089097)	1.122	0.262028
Lymph node metastasis[b]	0.626513 (0.171031)	3.663	0.000249
mRNA expression levels of EVX1	0.006638 (0.004173)	1.591	0.111696

[a]Pathological Tumor-Node-Metastasis stage. [b]Positive or negative.

(c) Clinicopathological parameters and mRNA expression levels of GFRA1

	Coefficient (Standard error)	*Z*	*P*
Pathological TNM stage[a]	0.10370 (0.08922)	1.162	0.2451
Lymph node metastasis[b]	0.36454 (0.17060)	3.719	0.0002
mRNA expression levels of GFRA1	0.04746 (0.08963)	0.530	0.5964

[a]Pathological Tumor-Node-Metastasis stage. [b]Positive or negative.

(d) Clinicopathological parameters and mRNA expression levels of PDE9A

	Coefficient (Standard error)	*Z*	*P*
Pathological TNM stage[a]	0.103868 (0.088883)	1.169	0.242567
Lymph node metastasis[b]	0.629262 (0.170732)	3.686	0.000228
mRNA expression levels of PDE9A	0.009987 (0.007854)	1.272	0.203497

[a]Pathological Tumor-Node-Metastasis stage. [b]Positive or negative.

(e) Clinicopathological parameters and mRNA expression levels of TBX20

	Coefficient (Standard error)	*Z*	*P*
Pathological TNM stage[a]	0.09349 (0.09016)	1.037	0.2998
Lymph node metastasis[b]	0.63752 (0.17365)	3.671	0.000241
mRNA expression levels of TBX20	0.69616 (0.39752)	1.751	0.079904

[a]Pathological Tumor-Node-Metastasis stage. [b]Positive or negative.
